# Allosteric Communication across the Native and Mutated KIT Receptor Tyrosine Kinase

**DOI:** 10.1371/journal.pcbi.1002661

**Published:** 2012-08-23

**Authors:** Elodie Laine, Christian Auclair, Luba Tchertanov

**Affiliations:** LBPA, CNRS - ENS de Cachan, LabEx LERMIT, Cachan, France; National Cancer Institute, United States of America and Tel Aviv University, Israel, United States of America

## Abstract

A fundamental goal in cellular signaling is to understand allosteric communication, the process by which signals originated at one site in a protein propagate dependably to affect remote functional sites. Here, we describe the allosteric regulation of the receptor tyrosine kinase KIT. Our analysis evidenced that communication routes established between the activation loop (A-loop) and the distant juxtamembrane region (JMR) in the native protein were disrupted by the oncogenic mutation D816V positioned in the A-loop. *In silico* mutagenesis provided a plausible way of restoring the protein communication detected in the native KIT by introducing a counter-balancing second mutation D792E. The communication patterns observed in the native and mutated KIT correlate perfectly with the structural and dynamical features of these proteins. Particularly, a long-distance effect of the D816V mutation manifested as an important structural re-organization of the JMR in the oncogenic mutant was completely vanished in the double mutant D816V/D792E. This detailed characterization of the allosteric communication in the different forms of KIT, native and mutants, was performed by using a modular network representation composed of *communication pathways* and *independent dynamic segments*. Such representation permits to enrich a purely mechanistic interaction-based model of protein communication by the introduction of concerted local atomic fluctuations. This method, validated on KIT receptor, may guide a rational modulation of the physiopathological activities of other receptor tyrosine kinases.

## Author Summary

The majority of functionally important biological processes are regulated by allosteric communication within individual proteins and across protein complexes. Receptor tyrosine kinases (RTKs) control signal transduction pathways and consequently represent a typical paradigm. The mutation-induced deregulation of RTK activity impairs crucial cellular physiological functions and causes serious human diseases. The present study focuses on the allosteric communication across the three-dimensional structure of the RTK KIT cytoplasmic region. Combining a mechanistic model of information transmission with the analysis of concerted local atomic fluctuations we examined and compared the communication profiles in the native and D816V-mutated proteins. This approach permitted to localize and visualize communication routes in the native KIT and revealed that these routes were disrupted in the mutant D816V. We proposed *in silico* mutagenesis as a mean to restore the communication detected in the native KIT. Our work sheds light on the allosteric communication in RTKs, a phenomenon playing an essential role in signaling pathways albeit experiments do not provide the atomic details of the path followed in going from one structural element to the other. A rational understanding of the molecular determinants underlying the effects of disease-related kinase mutations may contribute to the improvement of targeted therapies.

## Introduction

Signal transduction in cells is regulated through complex networks of dynamical interactions between macromolecules. The proteins controlling these communication networks respond to changes in the cellular environment by switching between different conformational states [1]. A great number of proteins acting as ligand/substrate-dependent activators contribute to cell signaling pathways. Among such proteins, receptor tyrosine kinases (RTKs) play a leading role in the regulation of physiological processes crucial for cell survival, growth, proliferation and differentiation [2]. In general, the binding of a ligand to the extra-cellular region of RTKs induces receptor dimerization which in turn leads to the activation of the intracellular tyrosine kinase domain. All RTKs are ATP-binding proteins and catalyze the same reaction, i.e. the transfer of the 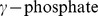 to specific tyrosine sites. They thereby trigger multiple signaling cascades via the recruitment of enzymes and adaptor proteins [3]–[5].

Kinase domains are essentially molecular switches that can adopt at least two extreme conformations – the “on” and “off” states, corresponding to maximum and minimum protein activity. The catalytically efficient “on” conformation is very similar (conserved) among all kinases. The “off” state (inactive) is not subject to the biological and structural constrains that the active state must supply, and consequently different types of kinases have developed distinct (divergent) conformations. In the native state, RTKs preexist in several conformations ranging between these two extreme ones. The equilibrium between the various conformational populations can be displaced by the binding of an extra-cellular ligand or an inhibitor, phosphorylation events or point mutations [6]. In particular mutations are mainly responsible for the deregulation of RTK activity, provoking various forms of cancer, inflammatory diseases (e.g. arthritis) and neuronal disorders (e.g. Alzheimer's pathology) [7].

The binding of a ligand/substrate/inhibitor, a point mutation, the modification of the amino acids ionization state or environmental changes (pH, temperature, concentration, ionic forces) can be considered as perturbations of a biological object and described in terms of signal propagation theory and molecular dynamics [8]–[12]. The propagation of a perturbation signal across a protein three-dimensional structure relates to the concept of allosteric coupling or communication. The increasing amount of high-quality experimental data provides evidence that allosteric communication is a general phenomenon observed in the majority of proteins [13]. Allosteric regulation is at play when a given perturbation at a specific site of a protein alters the conformational and/or thermodynamic state of a spatially distinct site in the same molecule.

According to the classical view [14], [15], allosteric coupling occurs as an outcome of a network of interactions that physically link the coupled sites – two-state structural-based signal transmission through a unique pathway. However, multiple experimental evidences have shown that allosteric coupling can be mediated solely by transmitted changes in the protein dynamics/motions [6] as a consequence of a re-distribution of the protein conformational populations [16]. This statement suggests the existence of multiple potential communication tracks with a preferred pathway under a given condition. Attempts were recently made to classify protein allosteric effects and illustrate those using typical examples [17]. Such examples reflect the dual nature of allosteric coupling, which can be manifested in the form of a global conformational change or the modification of local atomic fluctuations. In either case, information transmission may take place through well-structured connectivity pathways or multiple dynamic micro-pathways in the protein residue network [18], [19].

A number of *in silico* techniques aiming at predicting connectivity pathways that mechanically transmit allosteric interactions have been developed, based on evolutionary conservation information [20]–[22], native contacts within the protein residue network [23], [24] or dynamical correlations from molecular dynamics (MD) simulations [25]–[29]. Some efforts have also been made to identify specific sites in proteins that are able to accumulate energy in response to perturbations, even occurring at distant locations [11], [30], [31]. These approaches were developed with the aim of providing a rational understanding of various allosteric effects in divergent proteins and their complexes.

Mutation-driven allosteric regulation bears a particular interest due to the importance of mutations in clinical and pharmacological research. Clarkson and co-authors have observed two types of propagation responses to point mutations in serine proteinase eglin c, a small globular protein [32]. One type of propagation response was discovered in the form of contiguous pathways of dynamic changes and the other as dispersed changes associated with subtle protein backbone deformations. Following these observations, Schrank and co-authors were able to design adenylate kinase mutations that impact distant functional sites only through the modulation of the protein atomic fluctuations [33]. MD simulations of the native and mutated forms of the cytoplasmic protein kinase ABL and the EGF receptor probed the global allosteric changes induced by oncogenic mutations [34]. In these cases, the allosteric communication was described in terms of a dynamic coupling between structurally rigid and conformationally adaptive segments [25], [35]. It was supposed that these structural elements form a dynamic network of interacting functional regions that control the long-range inter-domain communication and allosteric activation in protein kinases.

**Figure 1:**
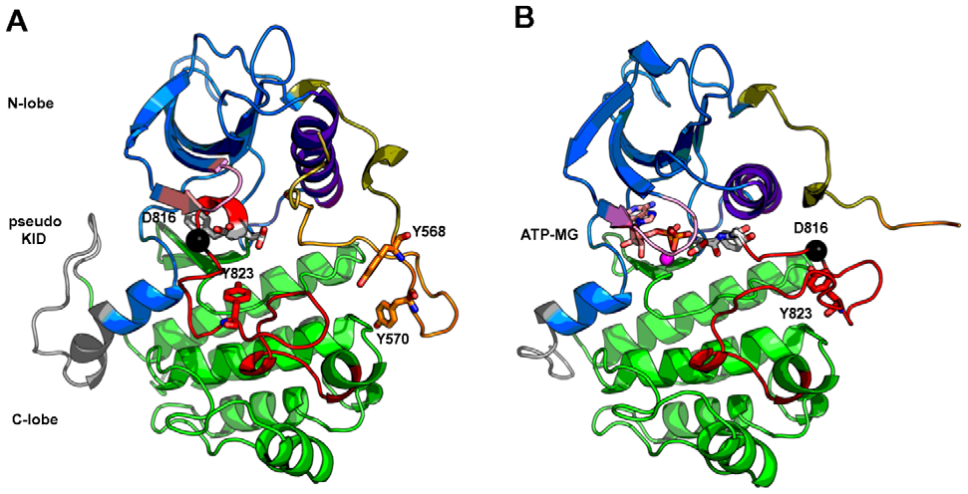
Structure of KIT cytoplasmic region. The crystallographic structures of wild type KIT cytoplasmic region in the inactive auto-inhibited (**A**, PDB code: 1T45 [70]) and active (**B**, PDB code: 1PKG [54]) states are represented as cartoons. The N-terminal proximal lobe (N-lobe) is in blue, the C-terminal distal lobe (C-lobe) is in green and in-between the truncated kinase insert domain (pseudo-KID) is in gray. The four fragments composing the juxta-membrane region (JMR) are delineated by their colors: JM-Proximal (JM-P) in yellow-orange, JM-Buried (JM-B) in bright orange, JM-Switch (JM-S) in orange and JM-Zipper (JM-Z) in deep olive. The activation loop (A-loop) is in red – its DFG motif highlighted in sticks with carbon atoms in white, the C-helix is in purple and the glycine-rich loop is in pink. Tyrosines representing phosphorylation sites are drawn as sticks: Y568 and Y570 (JMR), Y823 (A-loop). The location of the mutational hot spot residue 816 is indicated by a black sphere depicting the position of its 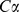 atom.

We are studying the type III RTK family, which includes the stem cell factor (SCF) receptor KIT, along with PDGFR, Flt3 and FMS. Upon the binding of the physiological ligand SCF, KIT ectodomain undergoes a conformational change that leads to dimerization and in turn activation of the intra-cellular protein kinase domain (PTK). The activation of KIT cytoplasmic region is characterized by a large conformational transition, that involves the detachment of the autoinhibitory juxtamembrane region (JMR, residues 544–581) from PTK, the opening of the activation loop (A-loop, residues 810–835) away from the ATP binding site and the structural rearrangement of regions such as the C-helix (residues 631–647) and the glycine-rich loop (residues 596–601) (**Figure 1**). Point mutations localized in the JMR or the PTK of KIT were identified in various forms of cancer (gastro-intestinal stromal tumors GISTs, acute myeloid leukemia AML, mast cell leukemia MCL, germ cell tumors…) and mastocytosis [36]–[39]. In particular, the substitution of the amino acid in position 816 located in the A-loop (indicated as a black sphere on **Figure 1**) from aspartate (D) to valine (V) is an activating mutation associated with mastocytosis and cancer and is archetypal of the resistance to anti-cancer drugs such as Imatinib (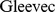
®) [40], [41].

As was evidenced by X-ray data, the overall fold of KIT kinase domain is not significantly altered by point mutations in position 816 [42]. However, we have previously demonstrated the impact of the D816V mutation on the structure and dynamics of two regulatory segments of the KIT cytoplasmic region [43]. First, we have shown that D816V induces the partial unfolding of the small 817–819 helix in the A-loop, a local effect prompted by the loss of the negative capping of the helix by the aspartate in position 816. The second and noteworthy event consisted in the stabilization of the anti-parallel 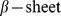 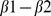 in the JM-Switch fragment of the JMR located more than 15 Å away from the mutation site in the inactive state (**Figure 1 A**). This long-range effect led to a notable secondary structure reorganization of the JMR accompanied by a tertiary restructuring that resulted in an axial repositioning of the JM-Switch respectively to PTK [43]. Such long distance structural effect is indubitably a manifestation of allosteric intra-molecular communication in KIT.

The analysis of the interaction network between the JMR and PTK regions of KIT gave preliminary insights into the non covalent contacts alterations induced by the 816V mutation [43]. In the present work, we described the allosteric communication in the native and D816V-mutated KIT in terms of information transmission. We evidenced (i) a well-established communication between the activation loop (A-loop) and the distant juxtamembrane region (JMR) in the native protein, (ii) the disruption of such communication in the mutant produced by the oncogenic mutation D816V and (iii) the restoring of the communication by *in silico* mutagenesis through a counter-balancing mutation D792E. The communication patterns observed in the native and mutated KIT correlated with their structural and dynamical properties. Particularly, the long-range effect of the D816V mutation on the JM-Switch fragment of the JMR was completely disappeared in the double mutant D816V/D792E. The JMR structure in the double mutant was found very similar to that observed in the native KIT.

**Figure 2:**
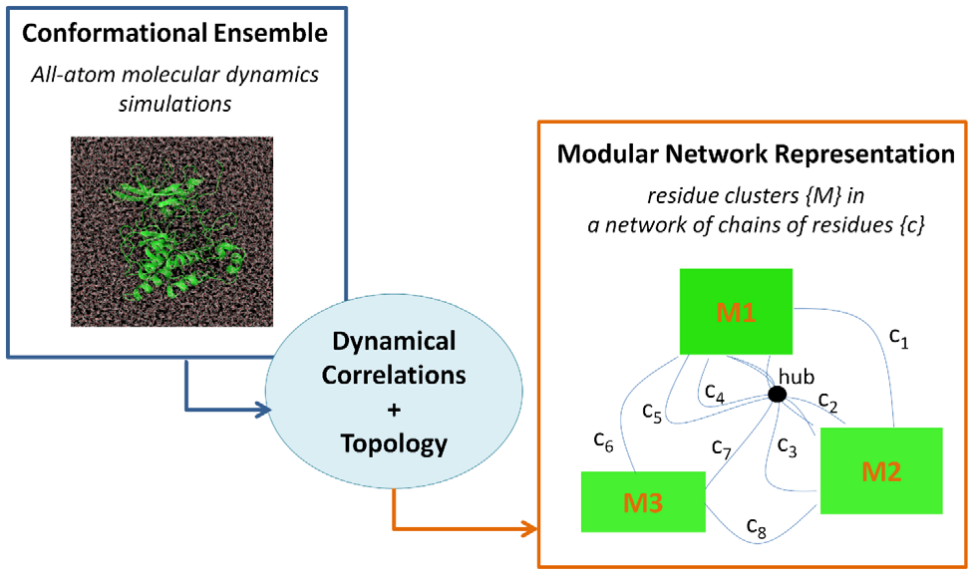
Schematic representation of the MOdular NETwork Analysis (MONETA) general principle. From a protein conformational ensemble the dynamical correlations and topology are calculated and used to build a modular network representation composed of clusters of residues 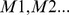 and chains of residues 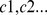. Information is propagated either within residue clusters which form modules or along chains which link individual residues. In our particular implementation of the method, residue clusters or modules are designated as *independent dynamic segments* as they represent the most striking features of the protein local dynamics. Chains of individual residues are designated as *communication pathways* as they represent well-defined connectivity pathways along which interactions can be mediated at long distances in the protein. Information is propagated through *independent dynamic segments* via the modification of the local atomic fluctuations and through *communication pathways* via well-defined interactions. Highly connected residues, at the junction of many pathways, can be considered as *“hubs”* in the protein network representation.

The accurate characterization of the allosteric communication in the different forms of KIT, native and mutants, was performed by using a modular network representation composed of *communication pathways* and *independent dynamic segments* (**Figure 2**). Such representation enriches a purely mechanistic model of protein communication based on well-defined interactions by the introduction of concerted local atomic fluctuations. The approaches were implemented in the form of a MOdular NETwork Analysis (MONETA) package coupled with the R software [44] and the python scripting interface of Pymol [45]. This package permits to efficiently build, analyze and visualize modular network representations of protein three-dimensional structures.

## Results/Discussion

### Computational modeling of allosteric communication across KIT

Although molecular insight has been gained for the precise assessment of the D816V mutation induced conformational changes in KIT kinase, the description of the allosteric communication taking place between the two functionally distinct and spatially distant receptor regulatory regions – the activation loop (A-loop) and the juxtamembrane region (JMR) – remains a challenge. In the present study, a two-component molecular model of intra-protein signal propagation was used to: (a) determine key residues or regions in KIT kinase involved in allosteric coupling, (b) perform a comparative analysis of wild type KIT and D816V mutant allosteric communication profiles, (c) propose a protein-based design to neutralize the long-range effect of D816V mutation on KIT structure and dynamics.

The allosteric communication across KIT tyrosine kinase was analyzed by using the conformational MD ensembles generated previously for the native (WT) and D816V-mutated (MU) KIT cytoplasmic region in the auto-inhibited inactive state [43]. Intra-molecular interactions and inter-residue dynamical correlations were computed from the MD trajectories and were used to identify chains of connected residues or *communication pathways* and clusters of locally coupled residues or *independent dynamic segments* (**Figure 2**).

The ability of KIT protein residues to communicate efficiently was evaluated by using the measure of communication propensity [9]. The communication between two residues is estimated as fast when their *commute time*, expressed as the variance of their inter-residue distance over long MD trajectories [25], [46], is small. We supposed that chains of residues interacting by pair and displaying high communication propensities between them would represent pathways of well-defined interactions through which information would be transmitted efficiently. We denote such chains of residues as *communication pathways* (*CPs*). Any two adjacent residues in the *CP* are connected by non-bonded interaction(s) and the *commute time* for transmitting information between any two members of a *CP* is below a given threshold (see Materials and Methods for a detailed description).

To identify regions of KIT that represented the most striking features of the protein's local dynamics, we used a statistical technique, local feature analysis (LFA) [47]. This formalism projects the correlation matrix computed from an MD trajectory in such a way that it reduces off-block diagonal correlations and identifies *n seed* degrees of freedom corresponding to 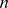 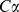 atoms or residues. Further, clusters of neighboring residues centered on the *n* selected *seed* residues and displaying concerted local atomic fluctuations were defined (see Materials and Methods for details on the implementation). Assuming that they represent regions of the protein that are independent in terms of their dynamical behavior, we denote such clusters of residues as *independent dynamic segments* (*IDSs*). *IDSs* are believed to play a crucial role in binding and/or in allosteric propagation by shifting or exchanging their energy content [11], [30], [31], [48].

*communication pathways* and *independent dynamic segments* can be considered as two different media through which a given perturbation is likely to be propagated either mechanically via well-defined interactions (*CPs*) or via concerted local atomic fluctuations (*IDSs*). The combination of these two types of analyzes provided a useful two-component modeling framework for the characterization of the allosteric communication in KIT tyrosine kinase in the wild type and mutated forms. Importantly, such framework permits to go beyond a purely mechanistic model of signal propagation in proteins and to account for the duality inherent to the manifestations of allosteric coupling.

### Key structural elements of KIT serve as communication *hubs*

**Figure 3:**
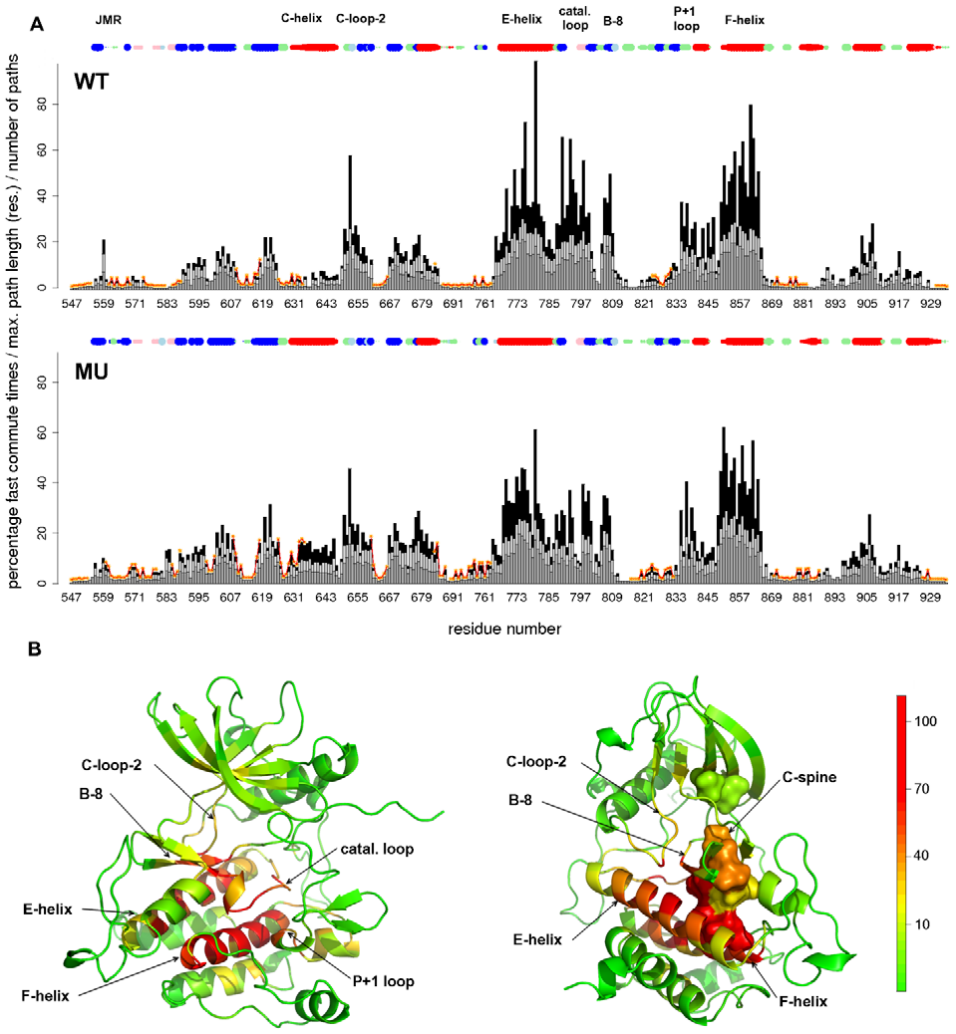
Characteristic features of the *communication pathways* generated in KIT cytoplasmic region. (**A–B**) Barplots giving the characteristic features of the paths generated from each residue of the wild type (WT, top panel) and the mutant (MU, bottom panel). The percentage of fast *commute times* (lower than 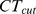, see Materials and Methods), the maximum path length (in residues) and the number of paths are represented as dark gray, light gray and black bars, respectively. *Independent dynamic segments* are traced in orange. The secondary structure elements are depicted as juxtaposed points at the top of each graph, color-coded as follows: 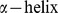 in red, 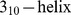 in pink, parallel 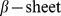 in light blue, anti-parallel 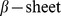 in blue, turn in green and coil in white. The size of the point is proportional to the value of the secondary structure type occupancy over the MD simulations. (**B**) Structural mapping of the communication efficiency of KIT residues in WT presented in two different views. The average MD conformations are represented as cartoons and residues are colored from green through yellow to red according to their communication efficiency, estimated as the sum of their percentage of fast *commute times*, maximum path length (in residues) and number of paths. The loop following the C-helix (C-loop-2, residues 649–655), the E-helix (residues 764–785), the catalytic loop (residues 790–797), the 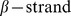 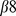 (residues 804–808), the loop immediately following the A-loop (P+1 loop, residues 835–843) and the F-helix (residues 850–865) are labeled. The C(catalytic)-spine residues, as defined in [52], are shown as a partial molecular surface on the right panel.

*communication pathways* were systematically generated from each residue of both wild type (WT) and D816V-mutated (MU) KIT proteins. The per-residue percentage of fast *commute times*, *CP* maximum length and number of *CPs* are reported on **Figure 3 A**. Highly connected residues or *hubs*, located at the crossroad of many *CPs*, display about 20% of fast *commute times*. Structural mapping of *CPs* characteristic features brings out the structural fragments of KIT that present such *hubs* (**Figure 3 B**): the loop following the C-helix (C-loop-2, residues 649–655), the E-helix (residues 764–785), the catalytic loop (residues 790–797), the 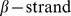 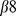 (residues 804–808), the loop immediately following the A-loop (P+1 loop, residues 835–843) and the F-helix (residues 850–865). Moreover, a dense network of *CPs* links three of these structural fragments, namely C-loop-2, the E-helix and 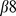 (**Figure S1**). In addition, residues L678, I798, L799, L800, F858 and L862 that compose KIT catalytic spine (see below) also display high communication capabilities (protein partial surface shown on **Figure 3 B**, right panel).

The biological significance of these structural fragments was previously reported in the literature. Namely, C-loop-2 was identified as part of a “molecular brake” crucial for RTKs inactive state stabilization [49]. It was also suggested that C-loop-2 may control the long-range inter-monomer coupling in the activation complexes of the receptor tyrosine kinase EGFR [25]. The E-helix was found to stabilize the ATP binding pocket in a large number of kinases through its interactions with the 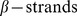 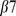 and 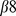 [50]. The catalytic loop is highly conserved in terms of structure and sequence among kinases and acts as a cornerstone of the active site [51]. The P+1 loop contains a conserved APE motif (residues 837–839) [51]. The F-helix was shown to play an important role in the dynamic assembly of protein kinase active structure, serving as an anchor for two hydrophobic spines that traverse both lobes of kinases, the C(catalytic)-spine and the R(regulatory)-spine [52]. Furthermore the integrating role of the F-helix for allosteric communication in the cytoplasmic kinase ABL and in the RTK EGFR was recently pointed out [25].

Consequently, the structural fragments of KIT identified by our *CP* analysis as presenting communication hubs were previously reported as playing crucial functional roles in the activation/deactivation mechanisms of other receptor tyrosine kinases or cytoplasmic kinases.

### D816V mutation induces a reshaping of KIT communication profile

Although the communication profiles of WT and MU KIT share a similar global shape (**Figure 3 A**), a number of dissimilarities are observed. For example, certain residues display shorter and less probable *CPs* in MU compared to WT. This deterioration of communication efficiency is particularly evident for residue V559 of the JMR, residue N652 of the C-loop-2, several residues in the E-helix (residues 764–785), the catalytic loop residues (residues 790–797), the two residues 808–809 between the 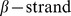 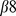 and the A-loop and the residues of the P+1 loop that follows the A-loop. In terms of communication, residues N652 of the C-loop-2 and residues A794, A795 and N797 of the catalytic loop are involved in dense networks of *CPs* that span across both lobes of the protein in WT, whereas they communicate efficiently with residues located only within one lobe in MU, the N-lobe for N652 and the C-lobe for the catalytic loop residues. This very reduced number of *CPs* may indicate a break in the communication between the N- and C-lobes of KIT cytoplasmic region induced by the mutation D816V.

By contrast several residues in the N-lobe display slightly increased communication efficiency in MU compared to WT (**Figure 3 A**). They are located after the JMR (583–584), in the loop preceding the C-helix (residues 625–630) and in the C-helix (residues 631–647). These residues display very poor communication capability in WT while they participate in *CPs* that extend along the C-helix or across the 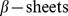 of the N-lobe in MU. This increased communication of specific regions of the N-lobe is accompanied by a significant reduction of their atomic fluctuations upon D816V mutation.

Consequently, the comparative analysis of WT and MU KIT communication profiles reveals that the mutation D816V alters the allosteric communication between the N- and C-lobes of KIT protein, while it favors the emergence of dense and rather localized communication networks within the N-lobe.

### Regulatory segments of KIT display independent dynamic behaviors

**Figure 4:**
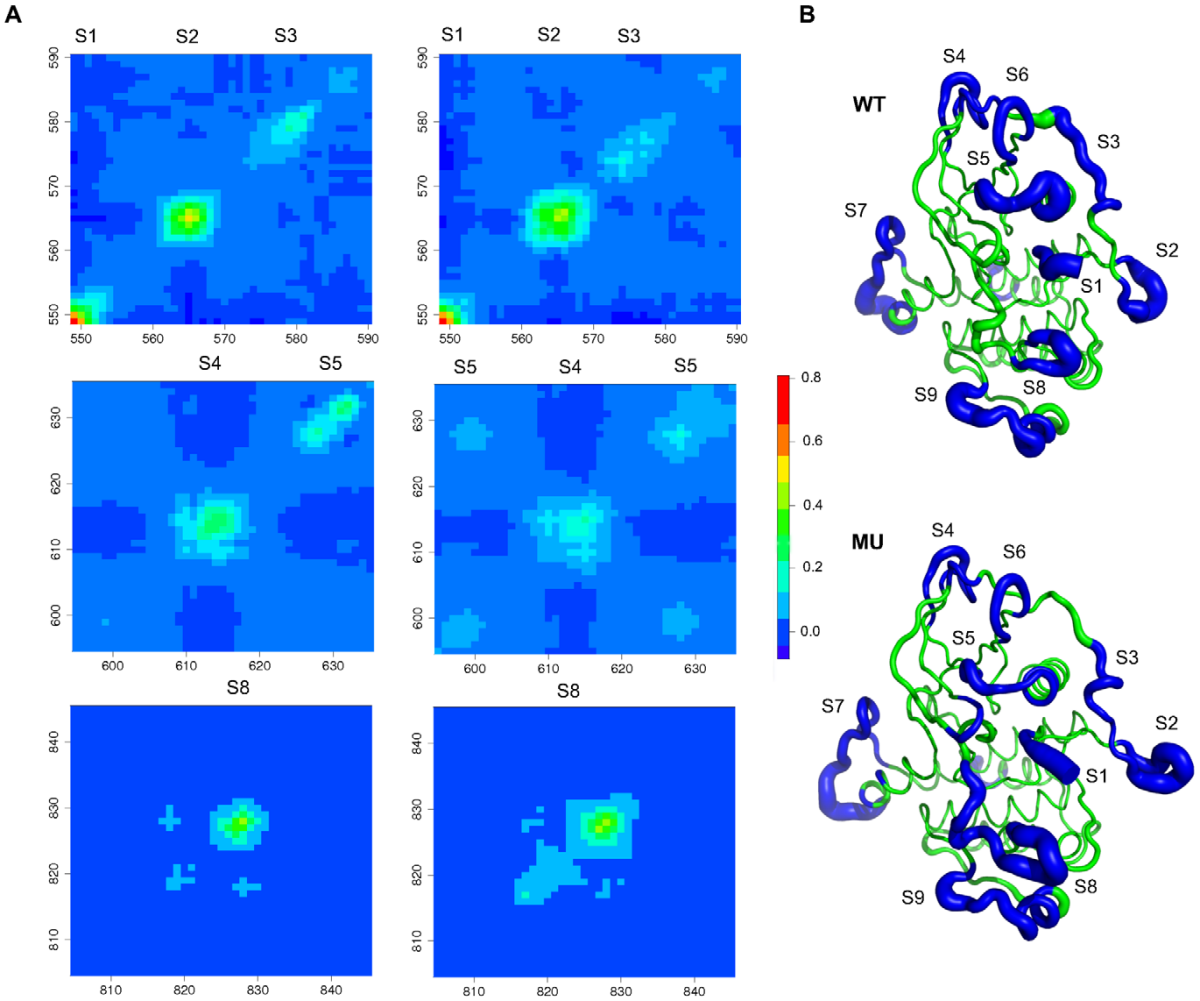
*Independent dynamic segments* identified in KIT cytoplasmic region. (**A**) Residual correlation submatrices corresponding to the JMR (top panels, residues 549–590), the N-lobe (middle panels, residues 595–635) and the A-loop (bottom panels, residues 805–845) regions in WT (on the left) and MU (on the right). The color code indicates the degree of correlation from negative values in blue to high positive values in red. The corresponding *independent dynamic segments S1*, *S2*, *S3*, *S4*, *S5* and *S8* are labeled. (**B**) Structural mapping of the *independent dynamic segments* (*IDSs*) identified in WT and MU. The average MD conformations are represented as tubes: *IDSs* are highlighted in blue while the remaining architectural core of the protein is in green. The size of the tube is proportional to the by-residue atomic fluctuations computed on the backbone atoms. The *IDSs S1* to *S9* are labeled.

*Independent dynamic segments* were identified in both WT and MU proteins. First, a principal component analysis (PCA) was performed on the 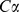 motions of KIT cytoplasmic region. The first 18 and 19 PCA modes were found sufficient to describe the essential dynamics of WT and MU, respectively. They were consequently retained to apply the subsequent local feature analysis (LFA) [47]. The LFA formalism selected 18 and 19 *seed* residues representative of the most striking features of WT and MU local dynamics. These residues were mostly located in KIT flexible regions: the N- and C-terminal extremities, the JMR, the truncated kinase insert domain (pseudo-KID), some solvent exposed loops in the N- and C-lobes and the A-loop (**Figure 4**). A similar localization of *seed* residues was also observed in previously reported studies [47], [53]. In each form, WT and MU, 10 *independent dynamic segments*, which we refer to as *Si*, 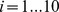, were then defined around these *seed* residues (**Table S1**).

*IDSs* represent about one third of the total number of KIT residues and their by-residue atomic fluctuations account for 56% and 60% of the total atomic fluctuations for WT and MU, respectively. *IDSs* are essentially composed of residues displaying fast communications only with neighboring residues along the sequence in the [i−4; i+4] range: no or very few *communication pathways* (*CPs*) were generated from residues contained in the *IDSs* (traced in orange on **Figure 3 A**). This observation is consistent with *IDSs* displaying dynamics that are minimally coupled to the other regions of the protein. Noticeably, the protein segments known to participate in KIT activation as key regulatory elements contain *IDSs* (**Figure 4**), namely the auto-inhibiting juxta-membrane region (JMR, residues 547–581), the C-helix (residues 631–647), the activation loop (A-loop, residue 810–835) and the G-helix (residues 877–885) that serves as a platform for the peptide substrate binding in KIT active structure [54].

The functional roles of these regions were previously characterized in different contexts of kinase-mediated physiological processes. The juxtamembrane region whose autoinhibitory function is specific to type III RTKs (KIT, PDGFR, C-FMS and FLT3) has also been shown to play crucial roles in the dimerization and activation mechanisms of other RTKs (EGFR, HER4) [55], [56]. The C-helix is involved in the R(regulatory)-spine [52] and has been emphasized as mediating conformational changes in the inactive-to-active transition of several kinases [55]–[59]. The importance of the A-loop conformation and its dynamics for kinases activity in general has been largely documented [4]. Although the G-helix function has not been characterized in details, recent experimental studies have demonstrated the essential roles of its mobility for several kinases [60], [61].

Consequently, the regulatory segments of KIT identified by our LFA analysis as containing or representing independent dynamics segments were previously reported as the elements mediating conformational changes associated with the activation/deactivation of other receptor tyrosine kinases or cytoplasmic kinases.

### D816V mutation modulates KIT local atomic fluctuations

*IDSs* are overall located in the same regions of KIT in WT and MU (**Figure 4**). However the boundary lines of the *IDSs* vary between the two proteins. The principal differences are observed for the *IDSs S2* and *S3* in the JMR (**Figure 4 A**, top panels), *S5* in the N-lobe (**Figure 4 A**, middle panels) and *S8* in the A-loop (**Figure 4 A**, bottom panels). Particularly, in the A-loop of WT, *S8* comprises residues 824 to 831 whereas in MU it expands toward the position of the mutated residue V816 and includes a large part of the A-loop (residues 816–832) (**Figure 4 B**). This expansion of the *IDSs S8* is a consequence of increased LFA correlations between the neighboring 816–823 and 824–831 fragments (**Figure 4 A**, bottom panels). Furthermore, the residues in this sequence (from 816 to 832) display an overall increase of atomic fluctuations in MU (2.57 Å
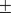
0.90 Å) compared to WT (1.73 Å
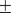
0.72 Å). In the JMR, the position of *S3* in KIT sequence is shifted from residues 574–581 in WT to residues 571–577 in MU (**Figure 4 B**). This shift corresponds to a change in the atomic fluctuations profile of the JMR residues. As a consequence *S2* and *S3* immediately follow each other in MU (**Figure 4 A**, top panels). These two *IDSs* correspond well to the structural definitions and morphological roles of the JM-Switch and JM-Zipper fragments respectively. In the N-lobe of WT, the *IDS S5* matches the highly flexible loop preceding the C-helix (residues 626–633) while in MU it is slightly expanded (residues 625–635) and also includes residues from the glycine-rich loop (residues 598–601) (**Figure 4 B**). This expansion is documented by off-diagonal correlations between the glycine-rich loop and the loop preceding the C-helix in MU (**Figure 4 A**, middle panels) and by a considerable decrease of the atomic fluctuations of residues 626–633 in MU (1.41 Å
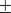
0.41 Å) compared to WT (2.34 Å
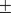
0.48 Å).

This comparative analysis of WT and MU local dynamics reveals that the D816V mutation influences the local atomic fluctuations of several structural fragments of KIT protein. In particular the JM-Switch, JM-Zipper, C-helix, P-loop and A-loop residues display more concerted atomic fluctuations in MU compared to WT. The A-loop dynamics changes indicate that the perturbation taking place in position 816 propagates to the downstream 817–819 helix, 820–823 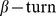 and 824–832 anti-parallel 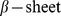. The apparition of concerted dynamics between the glycine-rich loop and the C-helix of mutated KIT may influence the protein activation as the glycine-rich loop normally helps positioning ATP [54].

The independent analyzes performed on KIT cytoplasmic region, one based on communication propensity and the other based on local dynamic features, provide a comprehensive two-component modeling framework for the rational description of information transmission throughout KIT structure. First, *communication pathways* enabled to identify residues or structural elements of KIT that serve as communication hubs in the protein residue network. Second, *independent dynamic segments* matched regulatory segments whose plasticity and structural rearrangements are essential for the inactive-to-active transition of KIT cytoplasmic region, as it has been reported in the literature.

Moreover the classification proposed here of the different regions of KIT according to their role highly connected *hubs* or locally correlated clusters in the protein allosteric communication can be related to recently published work identifying structurally rigid (minimally frustrated) and plastic (locally frustrated) clusters of residues in kinases [35]. Therefore, our analysis together with the reported data contribute to bring out general trends governing allosteric communication and structure assembly in this protein family.

### Localization and visualization of site-to-site communication between the A-loop and the JMR regions of KIT

In this section, we combined the definition of the *communication pathways*, *CPs*, and the characterization of *independent dynamic segments*, *IDSs*, to build an integrated modular network representation of KIT cytoplasmic region. We used such representation to localize and visualize the key factors governing the allosteric communication in KIT protein.

**Figure 5:**
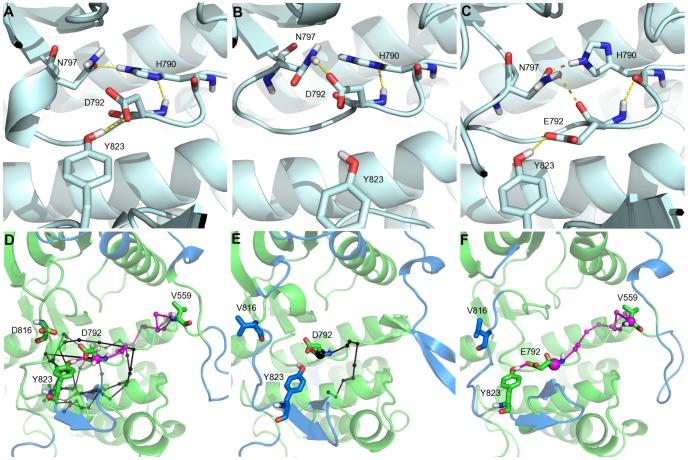
Interaction network and modular network representation in KIT cytoplasmic region. **Top**: The interaction network between the A-loop residues Y823 and the catalytic loop residues H790, D792 and N797 is depicted for wild type KIT (A), the D816V mutant (MU) (B) and the D816V/D792E double mutant (C). The average MD conformation is represented in pale cyan cartoons. H-bonds are displayed when their occupancy lies above 50% of the simulation time. **Bottom**: The modular network representations of the wild type KIT (**D**), the D816V mutant (**E**) and the D816V/D792E double mutant (**F**) built by MONETA are depicted, focusing on the JMR, catalytic loop and A-loop regions. The average MD conformation is represented in transparent cartoons. *Independent dynamic segments* are highlighted in blue. *Communication pathways* generated from residue in position 792 are displayed as chains of small black spheres connected together by black lines. Within a given path, each residue member is illustrated by up to three spheres corresponding to: the atom closest to the preceding residue in the path, the 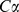, the atom closest to the following residue in the path. The initial residue is highlighted by a bigger sphere centered on its 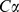. The path linking the A-loop and the JMR through the catalytic loop is highlighted in magenta. Residues D/V816 and Y823 in the A-loop, D/E792 in the catalytic loop and V559 in the JMR are highlighted in licorice and labeled.

In WT, the *IDS S8* localized in the A-loop was defined from residue 824 to 831. The analysis of non-covalent interactions between the A-loop and the other regions of the protein shows that an H(hydrogen)-bond between the hydroxyl oxygen atom of Y823 (A-loop), immediately preceding *S8*, and one of the carboxylate side-chain oxygen atoms (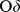) of D792 (catalytic loop) is formed during more than 95% of the simulation time (**Figure 5 A**). Moreover, residues D792, H790 and N797 of the catalytic loop are involved in a cyclic H-bond pattern to form a local interaction network that is very stable along the MD trajectories: the occupancies for the 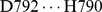 and 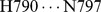 H-bonds are of 95% and 93% of the simulation time. These residues are also located at the crossroad of numerous *CPs*. In particular, one *CP* reaches residue V559 that immediately precedes the *IDS S2* in the JMR, as illustrated on **Figure 5 D**. Consequently in WT, information from the A-loop is transmitted to the JMR through the catalytic loop, with Y823 as a pivotal residue in this communication.

Upon D816V mutation, as we previously put in evidence [43], the small 817–819 helix shows partial unfolding. This structural change is associated with the expansion of the *IDS S8* from residues 816 to 832 and results in a shifted position of the residue Y823 unfavorable to the formation of the 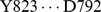 H-bond, whose occupancy consequently decreases by two folds down to less than 45% in MU (**Figure 5 B**). On the contrary, D792 rather interacts with N797 for more than 85% of the simulation time. Its H-bond contact with H790 side chain is perfectly preserved as in the WT (the occupancy factors are 98 and 95% respectively). This alteration of the catalytic loop local interaction network is accompanied by a drastic reduction of the catalytic loop residues capability to communicate efficiently with the distant KIT regulatory elements. As we mentioned above, several catalytic loop residues, including N797, are involved in dense networks of *CPs* than span both lobes of the protein in WT whereas the number of *CPs* is much reduced in MU and the remaining *CPs* are confined within the C-lobe. No established communication was observed between the residues D792 (catalytic loop) and V559 (JMR), which manifests a decoupling between the catalytic loop and the JMR in MU (**Figure 5 E**).

These observations suggest that the well-established allosteric communication in WT between the activation loop, A-loop, and the distant juxtamembrane region, JMR, channeled through the catalytic loop was disrupted by the oncogenic mutation D816V positioned in the A-loop. Consequently, we interpret the propagation of D816V effects between the A-loop and the JMR in terms of a communication break between the two remote sites. The structural reorganization of the JMR that we observed in MU [43] likely reflects this communication interruption. This interpretation is consistent with the results obtained by principal component analysis revealing a decoupling of A-loop and JMR motions in the mutant [43].

To further relate the observed changes in KIT allosteric communication with the thermodynamic properties of the protein, relative residue stability constants were calculated using the COREX/BEST algorithm [62], [63] for the WT and D816V-mutated forms. Consistent with the observed atomic fluctuations, a positive variation of 0.58 kcal/mol for the residues 816–832 (A-loop) and a negative change of −0.68 kcal/mol for the residues 560–570 (JM-Switch) stability constants were observed upon D816V mutation. These values may be indicative of the modulation of the energy landscape of KIT protein by the D816V mutation, an effect associated with the alternative communication in WT and D816V mutant unraveled here.

### Design of a double KIT mutant to achieve a counter-balancing/neutralizing effect

Our modular network analysis of KIT cytoplasmic region has revealed the crucial role of residues Y823 and D792 in the allosteric coupling/decoupling between the A-loop and the JMR. Considering the correlation observed between the occupancy value of the 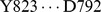 H-bond and the presence/absence of *CPs* between D792 (catalytic loop) and V559 (JMR) (**Figure 5 A–B,D–E**), we hypothesized that a way to re-establish the communication between the A-loop, the catalytic loop and the JMR in the D816V mutant would be to restore the 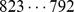 H-bond. To this aim, we substituted the aspartate (D) in position 792 by a glutamate (E), bearing the same charge and displaying a longer side chain. We anticipated that such replacement would facilitate the formation and stabilization of an H-bond with Y823 that adopted in MU an orientation unfavorable to such interaction. The *in silico*-designed double mutant D816V/D792E will be referred to as dbMU.

**Figure 6:**
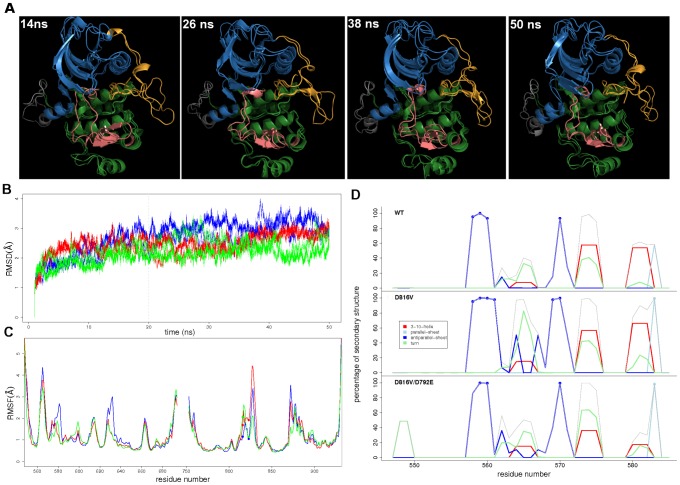
Structural and dynamics changes of KIT cytoplasmic region in the inactive auto-inhibited state upon mutations. (**A**) MD conformations taken at 14, 26, 38 and 50 ns of two 50-ns MD simulations of KIT D816V/D792E double mutant (dbMU), superimposed by pair and represented in cartoons: the JMR is in orange, the N-lobe in blue, the KID in gray, the A-loop in pink and the C-lobe in green. (**B**) Root mean square deviations (RMSD) in Å from the initial structures of KIT wild type (WT, in blue), D816V mutant (MU, in red) and dbMU (in green) computed on the backbone atoms along two MD replicas for each protein. (**C**) Backbone atomic fluctuations in Å of WT (in blue), MU (in red) and dbMU (in green). (**D**) Secondary structure assignments for the JMR in WT (upper panel), MU (middle panel) and dbMU (lower panel). The proportion of every secondary structure type is given as a percentage of the MD simulation time: 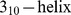 is colored in red, parallel 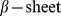 in light blue, anti-parallel 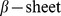 in dark blue, turn in green and the cumulative sum in gray. Residues forming 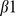 and 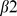 are indicated by blue points for each protein.

To characterize the structural and dynamical properties of the double mutant (dbMU), two 50-ns MD trajectories were produced using a protocol similar to that applied for WT and MU. The RMS deviations computed on the backbone atoms of dbMU (mean value, m. v., of 2.21 Å
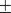
0.36 Å and max. at 3.48 Å) are comparable to those of WT and MU (m. v. of 2.64 Å
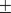
0.54 Å and max. at 4.04 Å, m. v. of 2.43 Å
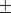
0.35 Å and max. at 3.36 Å, respectively) (**Figure 6 B**). Snapshots taken at 14, 26, 38 and 50 ns display the partial unfolding of the small 817–819 helix (**Figure 6 A**), the local effect of the D816V mutation also observed in MU (see Figure 4 in [43]). However the structure of the JM-Switch fragment of the JMR (residues 560–570) remains only partially ordered during the simulations (**Figure 6 A**), adopting in dbMU a conformation and a position similar to those observed in WT (**Figure 6 D**) and quite different from the well-structured axially positioned JMR of MU (see Figure 4 in [43]). The significant fold gain induced by the D816V mutation resulting in the stabilization of the extended anti-parallel 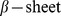 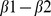 (residues 558–572) is not observed in the double mutant D816V/D792E.

To analyze the allosteric communication in the double mutant, a modular network representation was built. The characterization of *independent dynamic segments* in dbMU showed two distinct *IDSs* in the A-loop ranging from residue 814 to 821 and from residue 824 to 831 respectively, while one extended and homogeneous *IDS S8* from residues 816 to 832 was identified in MU. Moreover, the atomic fluctuations of residues 824–831 are considerably reduced in dbMU (max. of 2.11 Å at G827) compared to MU, while the flexibility of the residues 816–823 is higher in dbMU (max. of 2.62 Å at I817) than in MU (max. of 2.35 Å at I817) and in WT (max. of 1.81 Å at N819 in WT) (**Figure 6 C**). Furthermore, the analysis of non-covalent contacts in dbMU indicates an alternative local H-bond pattern that differs from those observed in WT and MU (**Figure 5 C**). First, the replacement of aspartate (D) to glutamate (E) at position 792 restored the 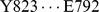 H-bond equivalent to that observed in WT (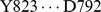). Second, E792 interacts stably (during 94% of the simulation time) with H790. Nevertheless the H-bond acceptor partner is changed from the nitrogen atom of histidine in WT and MU to the backbone carbonyl oxygen atom in dbMU. N797 residue in dbMU forms alternatively an H-bond either with H790 like in WT or with E792 like in MU with an approximately equivalent probability (45 and 51% respectively).

The observed H-bond pattern in dbMU shows a hybrid nature coming from the superimposition of two networks : the cyclic H-bond local interaction network observed in WT and the H-bond evidenced in MU. This hybrid H-bond interaction network in dbMU produces first the re-establishment of the allosteric communication between the A-loop and the JMR, through the catalytic loop as a channel (**Figure 5 F**) and second contributes directly or not to the conservation of the local structural changes induced by the D816V mutation (817–819 helix unfolding).

To elucidate the JMR thermodynamic properties in the double mutant, binding free energies of the JMR and its fragments to PTK were calculated. As we reported previously the JMR was more tightly attached to PTK in the WT KIT than in the D816V mutant [43]. Single-point MM-GBSA calculations (see thermodynamic cycle on Figure 3c in [43]) allowed to estimate the relative attachment of JMR to PTK in dbMU (**Table 1**). In spite of its tendency to overestimate the absolute values of free energy differences, the MM-GBSA method was often shown to reproduce well the correct free energy trends [64]. The global binding free energy change (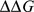) between dbMU and MU is of −50.62 kcal/mol, indicating that the additional mutation D792E restores the JMR attachment to PTK as observed in the wild type KIT. The entropic contribution, which reflects the conformational variability of the JMR, is more favorable in both WT and dbMU than in MU. In addition, the enthalpic contribution is also more favorable in dbMU compared to MU, suggesting that interactions between JMR and PTK are formed upon D792E mutation. Binding free energy changes computed for the different fragments of the JMR also show tighter attachment to PTK in dbMU compared to MU, except for the highly solvent-exposed JM-P. The computed values (**Table 1**) are very similar to those reported for the wild type and D816V-mutated form (Figure 3 in [43]). Consequently, these calculations reveal that the similar structural properties and dynamical behaviors displayed by the JMR in the wild type and D816V/D792E-mutated KIT forms correspond to nearly equivalent thermodynamic landscapes.

**Table 1:** Thermodynamic characterization of JMR in KIT wild type and mutants.

fragments	WT	MU	dbMU
	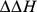	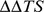	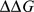
JM-P	−0.28	−15.39	15.11
JM-B	−1.04	15.01	−16.05
JM-S	−6.06	5.92	−11.98
JM-Z	−21.02	4.09	−25.09
JMR	−14.46	36.16	−50.62

Free energy 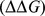, enthalpy 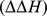) and entropy 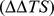 differences (in kcal/mol) for the binding of the JMR and its fragments JM-Proximal (JM-P, residues 547–552), JM-Binder (JM-B, residues 553–559), JM-Switch (JM-S, residues 560–570) and JM-Zipper (JM-Z, residues 571–581) to KIT kinase domain. Binding free energies were computed on the conformation after equilibration of KIT double mutant (dbMU) and were compared to the binding free energies reported for the wild type (WT) and the mutant (MU) in [43].

### The Y823-D792 link as a key factor in controlling KIT kinase activity

We interpreted the long-range structural and dynamic effects induced by the D816V mutation in terms of a communication disruption between two regulatory segments of KIT cytoplasmic region. Our results emphasize the importance of both Y823 and D792 residues in the allosteric communication between the A-loop and the JMR and consequently in the stabilization of the KIT native inactive state. In this section, we confront our results with experimental data available in the literature describing the biological importance of these residues.

Generally, Y823 stands as the phosphorylation site of KIT A-loop. However, recent *in vitro* characterization of Y823F-mutated KIT cytoplasmic region has shown that this mutant is auto-activated much faster than the wild type, while it remains very sensitive to inhibitors that target KIT A-loop inactive conformation [65]. These experimental observations support the hypothesis that the 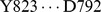 H-bond is a key factor in the control of the conformational switch between KIT inactive and active states. Indeed, any event ­ the attachment of a phosphate group on Y823 side chain or the substitution of Y823 by a phenylalanine ­ that shall weaken or completely abolish the interaction with D792 promotes KIT activation. Our molecular model of KIT allosteric communication provides additional useful insights on the mechanisms by which 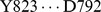 H-bond disruption may provoke the structural reorganization and repositioning of the remote JMR, facilitating its detachment from PTK – the triggering first step of the enzyme inactive-to-active state transition [43]. It should be noted that when Y823 is substituted by a phenylalanine, an 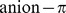 interaction can be established between F823 and D792 [66] that may still control the protein inter-conversion between inactive and active states.

Residue D792 located in KIT catalytic loop is highly conserved among protein kinases and was believed to act as a general catalytic base [67]. However, recent studies have shown non-consistency with such suggestion and it was proposed that this aspartate may rather assist substrate positioning or dissociation [68]. Mutation of this residue in several kinases significantly reduces the reaction rate but does not abolish activity [68]. Taking these data into consideration, we performed an *in silico* substitution of D792 by a glutamate in the structure of KIT active state (PDB id: 1PKG [54]), containing both ADP and peptide O-phosphotyrosine bound to the protein. The obtained active conformation of D792E mutant was found very similar to that of the wild type, suggesting that the protein active form can accommodate the longer side-chain of the glutamate with minimal structural rearrangement (**Figure S2**). Based on this observation, we supposed the D792E mutation would impact KIT conformational dynamics by inducing a partial counter-balance to compensate the destructive effect of the D816V mutation on the allosteric communication between two regulatory regions while preserving KIT enzymatic activities. This believable design would supply a framework for the *in vitro* comparison of the activation rates of the double mutant D816V/D792E, single mutant D816V and the native protein.

A number of other mutational hot spots were identified in KIT kinase [40], [42], [69], namely V560, V654, T670, D820, N822 and A829. V654 and T670 have been documented in samples from GIST patients exhibiting resistance against Imatinib [69]. V654, located in Cloop-2, generates 12 *CPs* spanning across both lobes of the protein. T670, often designated as the “gatekeeper” residue as it is located at the edge of the ATP binding site, participates in the network of *CPs* linking the C-loop-2, the E-helix and the 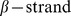 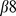. Therefore, our analysis suggests that these two mutational hot spots may be important for KIT allosteric communication as they likely contribute to mechanical information transmission. These suggestions agree with recent studies showing an important role for the “gatekeeper” residue T790 in the allosteric communication of the RTK EGFR [25].

As we showed, V560 of the JMR and A829 of the A-loop were identified as residues participating in *independent dynamic segments*, namely the *IDSs S2* and *S8*, respectively. D820 and N822 of the A-loop, together with D816, were found part of the *IDSs S8* only in the D816V-mutated KIT form. This finding suggests a common activating mechanism for the mutations of these residues. By a structural analysis of KIT inactive and active structures, we previously observed that D820 and N822 side chains form an H-bond that stabilizes a 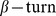 motif in the protein inactive state [43]. The clinically observed mutations D820A/E/G/Y and N822H/K are likely to disrupt the 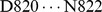 H-bond resulting in the unfolding of the 820–823 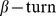 motif, which would produce a shifted position of Y823 unfavorable to the formation of the 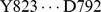 H-bond and hence to the establishment of the communication between the activation and catalytic loops. These findings depict a consistent description of the deregulation of KIT activity by oncogenic mutations which is in agreement with the available experimental data.

### Conclusion

We have studied the molecular determinants of the allosteric regulation of KIT receptor tyrosine kinase in the native form and two mutated forms, the oncogenic mutant D816V and the *in silico*-designed double mutant D816V/D792E. We were able to describe and modulate the communication between two remote principal regulatory segments, the activation loop (A-loop) and the juxtamembrane region (JMR). A strong correlation between such communication and the structural and dynamical features of the protein was established. The analysis was realized by a made-in-house computational tool MONETA based on a two-component modeling framework. This method, validated on receptor KIT, may guide a rational description and modulation of the physiopathological activities of other receptor tyrosine kinases altered by various perturbations such as phosphorylation events, substrate, ligand or inhibitor binding. The description of networks that represent only a local area (intra-molecular component) of vast communication pathways established between proteins, constitutes a first step toward an integrated description of signaling across different spatio-temporal scales, from intra-protein to cellular levels.

## Materials and Methods

Modular network representations of KIT cytoplasmic region were built and visualized with MONETA. The principle of the MONETA approach consists in building a modular network representation of the protein, composed of clusters of residues representing *independent dynamic segments* (*IDSs*) and chains of residues representing *communication pathways* (*CPs*) (**Figure 2**). The representation is derived from the topology of the protein and the inter-residue dynamical correlations calculated on a conformational ensemble, typically obtained by molecular dynamics (MD) simulations. *CPs* were generated based on the communication propensities [9] between all protein residues. *IDSs* were identified from local feature analysis (LFA) [47].

MONETA was implemented in the form of a package, coupled to the R software [44] and the python scripting interface of Pymol [45] – access to the package will be open by the end of 2012.

### Molecular dynamics trajectories production and analysis

Pre-processed data for wild type (WT) and D816V-mutated (MU) KIT were collected as described in [43]. The initial coordinates were taken from the crystallographic structure of the auto-inhibited inactive form of KIT (1T45, 1.90 Å resolution) [70]. *In silico* substitution of D816 into a valine (V) was performed using MODELLER 9v7 [71], [72]. Two 50-ns MD simulations were performed for each of the WT and MU proteins, with different starting velocities. Snapshots were written every 1 ps. A similar data generation and collection procedure was applied for the double mutant D816V/D792E (dbMU).

According to the RMSD profiles (see Figure 2 in [43] and **Figure 6 B**), the three systems WT, MU and dbMU took less than 20 ns to relax. Consequently, we retained the last 30 ns of each trajectory as the productive simulation time. This provided conformational ensembles of equal size (60 000 snapshots) for further statistical analysis. To assess the convergence of the data, the average time auto-correlation functions were computed the inter-residue 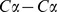 distances of WT, MU and dbMU:
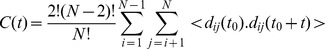
(1) where 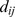 is the distance between the 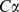 atoms of residue 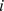 and residue 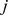, 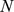 is the number of 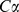 atoms and the normalizing pre-factor is the inverse of the total number of possible pairs of atoms from an ensemble of 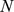 atoms. The obtained curves were fitted to exponential model functions 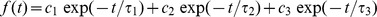 [73] using XmGrace [74]. Coefficients 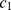 and 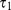 are given in **Table 2**.

**Table 2:** Coefficients 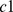 and 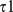 of the best fitted exponential curves to the average time autocorrelation functions of WT, MU and dbMU.

	WT	MU	dbMU
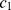	0.66	0.90	0.55
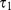	464.3	572.2	365.9

Correlations between the empirical functions and the fitted curves are within the 0.90–0.99 range.

Standard analyzes of the MD trajectories were performed with the *ptraj* module of AMBER 11 [75]. The DSSP method [76] was employed for secondary structure assignment within *ptraj*. Hydrogen bonds were detected between donors and acceptors (oxygen or nitrogen atoms) with a distance cutoff of 3.5 Å and no angle cutoff using the hydrogen bonding facility of *ptraj*.

Binding free energies of the JMR were evaluated using the MMGBSA method [77]–[79] implemented in AMBER 9 [80]. The binding free energies were evaluated on the equilibrated conformations of WT, MU and dbMU (single-point calculations). Details for the calculations were given elsewhere [43].

### Communication pathways

The concept of communication propensity [9] was used to identify *communication pathways* (*CPs*). The communication propensity of a pair of residues is inversely related to their *commute time* 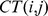, expressed as a function of the variance of the inter-residue distance [9]:
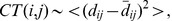
(2) where 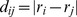 is the distance between the 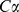 atoms of residue 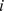 and residue 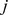, respectively. The smaller the variance the more efficient the communication between the two residues.

*commute times* can be calculated from the Kirchhoff matrix of an Elastic Network Model (ENM) [9] or from all-atom MD trajectories [25], [46]. In this work, *commute times* were computed between all protein residues from the last 30 nanoseconds of WT, MU and dbMU MD trajectories.

We describe *CPs* as chains of neighboring residues whose communication propensities between each other are high. Hence information is likely to be transmitted along *CPs* in short *commute times*. We generate *CPs* iteratively according to the following algorithm:

1. **Start** from a given residue 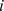

2. **Create** as many pathways as residue 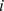
's *neighbors*
3. **Grow** each pathway, only if the newly added *neighbor* communicates efficiently with all the pathway members.

A residue 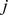 will be considered as a *neighbor* of residue 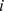 if residues 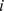 and 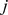: *(a)* are not adjacent in the sequence, *(b)* contact each other via non-bonded interactions and *(c)* communicate efficiently (*commute time* below 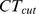). The way *communication pathways* are grown ensures that any two adjacent residues are connected by non-covalent interactions and that every residue in the *CP* is reachable from any other point in an equivalent short *commute time*.

Non-bonded interactions were recorded along the MD simulations using LIGPLOT [81]. Two residues were considered as interacting when they formed at least one non-bonded interaction for at least 50% of the simulation time. To discriminate between high and low communication propensities, ie between fast and slow *commute times* 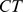, a threshold 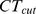 was chosen so that highly connected residues communicate efficiently with about 20% of the total number of residues in the protein, consistently with [46]. The threshold values were 0.09, 0.09 and 0.10 for WT, MU and dbMU respectively.

### Independent dynamic segments

We used a statistical technique called Local Feature Analysis (LFA) [47], [53] to identify *independent dynamic segments* (*IDSs*). Principal Component Analysis (PCA) was performed on the 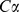 covariance matrices calculated from the 30 last ns of the MD trajectories of WT, MU and dbMU using *ptraj*. Among the 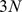 eigenvectors 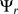 associated with eigenvalues 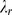, 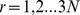, the first 19, 18 and 20 ones were sufficient to describe 80–85% of the total atomic fluctuations of WT, MU and dbMU respectively. These vectors were consequently used to apply the LFA formalism [47]. In brief, this formalism projects the correlation matrix in such a way that it reduces off-block diagonal correlations and identifies 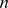 *seed* degrees of freedom corresponding to 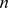 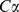 atoms or residues.

From the 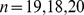 global PCA modes of WT, MU, dbMU, one can define 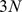 local LFA output functions 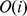 with minimum correlation [47]:
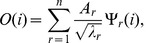
(3) where 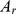 is the projection of the atomic fluctuations 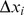 onto eigenvector 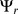: 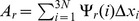. The residual correlations between LFA outputs are given by [47]:
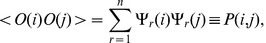
(4) so that in the limit 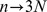 the LFA outputs are completely decorrelated by space: 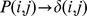.

Rather than computing all 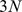 outputs 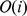, the sparsification algorithm described by Zhang and Wriggers [47] was applied using the statistics program R [44] to approximate the entire 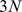 outputs with only a small subset of 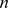 outputs 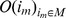. 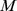 is the ensemble of 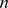 *seed* degrees of freedom that enable to reconstruct the outputs 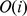 with minimal error. At each iteration of the sparsification algorithm, the outputs 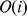 were reconstructed given the current set 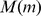 of 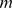 degrees of freedom and the reconstruction mean square errors 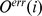 were evaluated. Out of the 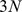 available degrees of freedom, the *seed* index displaying the maximum reconstruction error 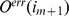 and not being already picked up was chosen as the 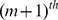 index into 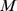. *Seed* indexes were added to 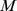 until 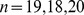 indexes were chosen, standing for 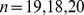 *seed* 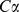 atoms or residues of WT, MU and dbMU respectively. Consequently, the obtained *seed* residues are representative of the most striking features of WT, MU and dbMU local dynamics.

The residual correlation 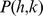 between residue 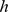 and residue 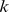 was evaluated as [47]:
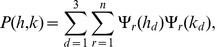
(5) where 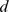 is the (x,y,z) coordinate index and 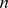 is the number of retained PCA modes.

We define *IDSs* as clusters of neighboring correlated residues whose centers are the *seed* atoms or residues identified by the LFA. Given a *seed* residue 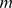, the corresponding *IDS* 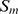 is extended progressively by adding neighboring residues in the protein structure three dimensional space as long as:
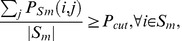
(6) where 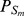 is the sub-matrix corresponding to the 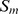 residual correlation pattern and 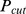 is the correlation threshold value. Distance matrices consisting of the average smallest distances between all residue pairs were computed using the *g_mdmat* program of GROMACS 4.5.3 [82]. Two residues i and j were considered close if the average smallest distance between them was lower than a given threshold 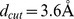. To discriminate between correlated and uncorrelated residues, a threshold value was arbitrary chosen as 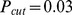. About 1.0–1.5% of all LFA cross-correlations lie above this threshold for WT, MU and dbMU.

## Supporting Information

Figure S1
***Communication pathways*** **linking the E-helix, the strand** 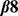 **and the C-loop-2 of KIT cytoplasmic region.** The average MD conformation of WT is represented as cartoons and residues are colored from green through yellow to red according to their communication efficiency, estimated as the sum of their percentage of fast *commute times*, maximum path length (in residues) and number of paths. *Communication pathways* generated from residue Q775 of the E-helix and reaching T806 of the strand 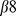 and N652 of the C-loop-2 are drawn as black spheres connected by black lines. The 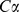 atoms of N652, Q775 and T806 are highlighted by bigger magenta spheres.(TIF)

Figure S2
***In silico*** **mutation D792E in KIT active structure.** The structures of KIT cytoplasmic region active state before (in green) and after (in blue) the substitution of D792 into E are superimposed and displayed in cartoon representation. The initial crystallographic structure (PDB id: 1PKG [54]) contains the ligands ADP and peptide O-phosphotyrosine. The mutation was performed *in silico* and followed by a slight minimization using the OPLS2005 force field in the Schrodinger suite [83], [84]. The ligand ADP, the phosphotyrosine (pTyr) and the residue D/E-792 are drawn in sticks and labeled. 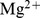 ion cofactors are shown as pink and magenta spheres, corresponding to the structure before and after the mutation respectively.(TIF)

Table S1 **LFA** ***seed*** **residues and associated residue clusters representing** ***independent dynamic segments*** **identified in KIT cytoplasmic region.** For each region of the protein, the *seed* residues identified by the LFA formalism are separated by commas and the associated *independent dynamic segments* are indicated in parentheses. The analysis was performed on the wild type (WT), mutant D816V (MU) and double mutant D816V/D792E (dbMU).
(PDF)
