# Supplementary figures and images for: Allosteric Communication across the Native and Mutated KIT Receptor Tyrosine Kinase
Source: PLoS Comput Biol. 2012 Aug 23;8(8):e1002661. doi: 10.1371/journal.pcbi.1002661 (PMC3426562; doi:10.1371/journal.pcbi.1002661)

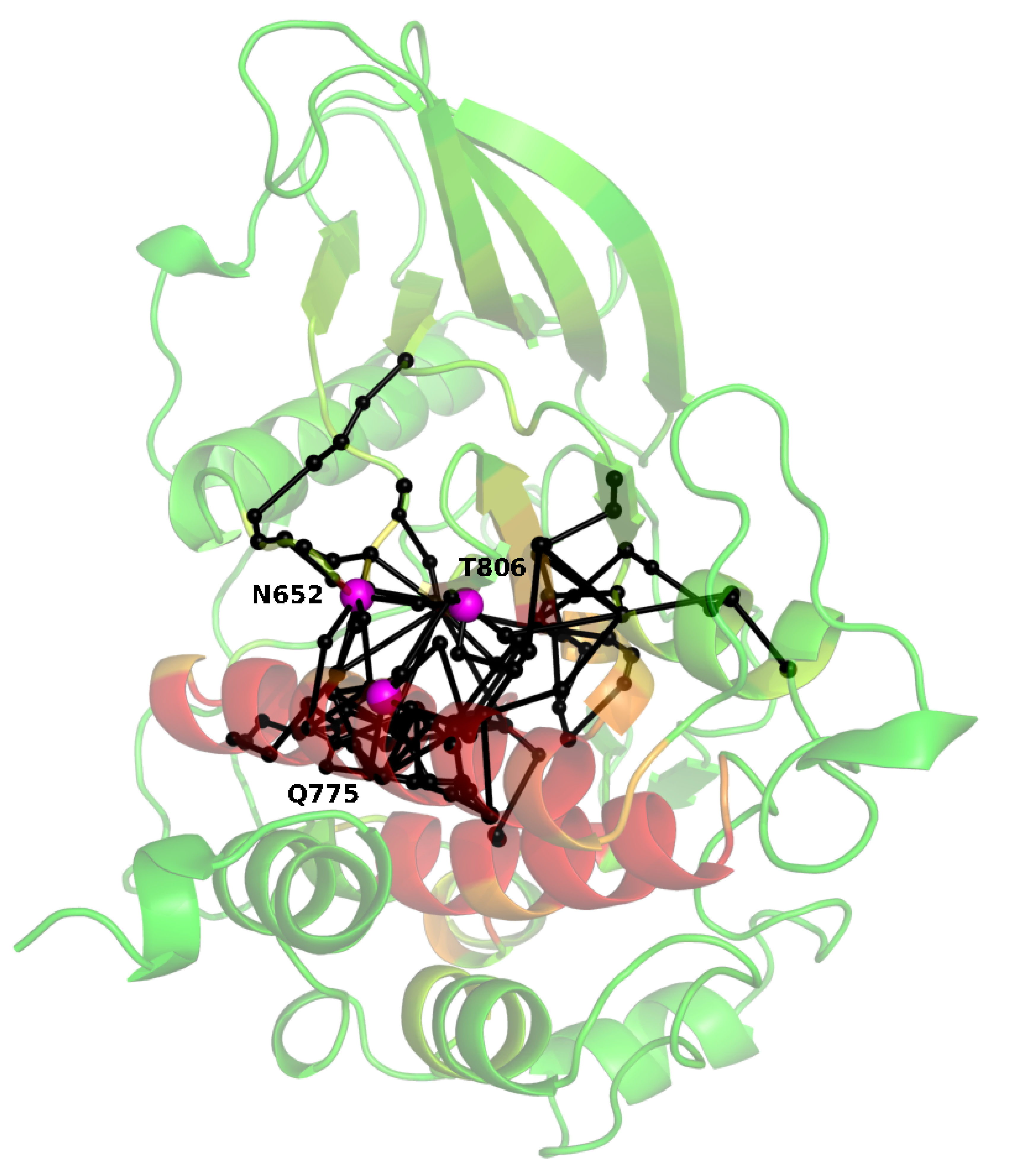

Supplement: Figure S1 — Communication pathways linking the E-helix, the strand and the C-loop-2 of KIT cytoplasmic region. The average MD conformation of WT is represented as cartoons and residues are colored from green through yellow to red according to their communication efficiency, estimated as the sum of their percentage of fast commute times, maximum path length (in residues) and number of paths. Communication pathways generated from residue Q775 of the E-helix and reaching T806 of the strand and N652 of the C-loop-2 are drawn as black spheres connected by black lines. The atoms of N652, Q775 and T806 are highlighted by bigger magenta spheres. (TIF) [file pcbi.1002661.s001.tif]

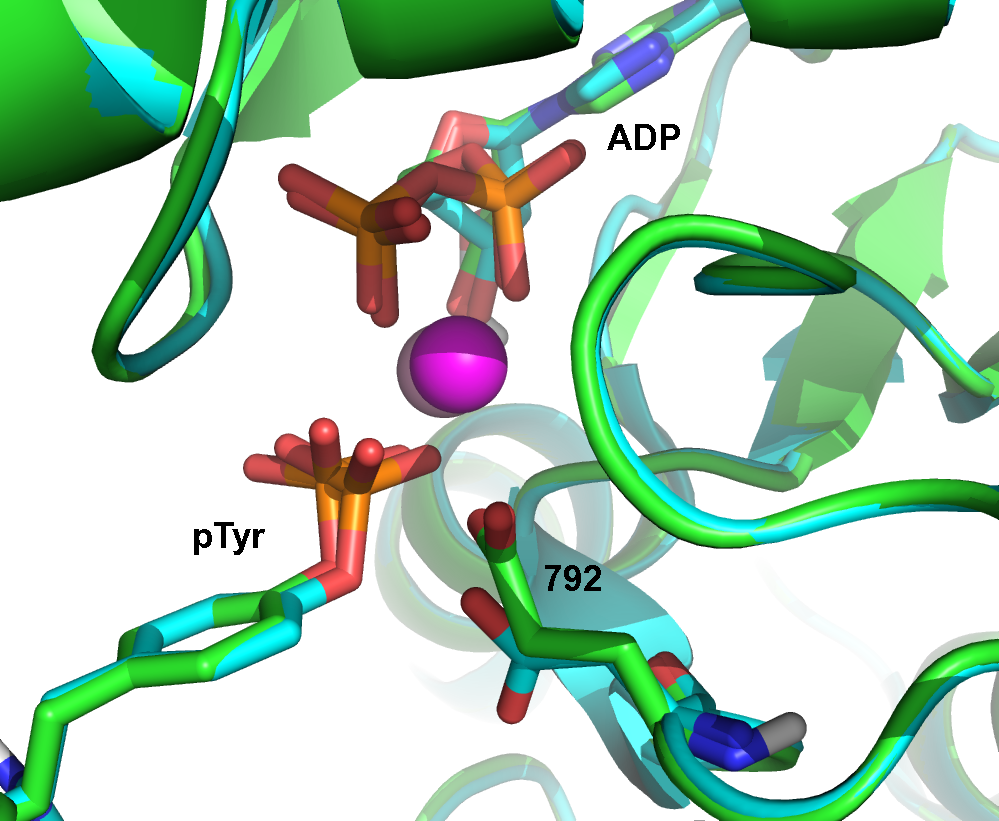

Supplement: Figure S2 — In silico mutation D792E in KIT active structure. The structures of KIT cytoplasmic region active state before (in green) and after (in blue) the substitution of D792 into E are superimposed and displayed in cartoon representation. The initial crystallographic structure (PDB id: 1PKG [54]) contains the ligands ADP and peptide O-phosphotyrosine. The mutation was performed in silico and followed by a slight minimization using the OPLS2005 force field in the Schrodinger suite [83], [84]. The ligand ADP, the phosphotyrosine (pTyr) and the residue D/E-792 are drawn in sticks and labeled. ion cofactors are shown as pink and magenta spheres, corresponding to the structure before and after the mutation respectively. (TIF) [file pcbi.1002661.s002.tif]
