# Supplementary material for: Allosteric Communication across the Native and Mutated KIT Receptor Tyrosine Kinase
Source: PLoS Comput Biol. 2012 Aug 23;8(8):e1002661. doi: 10.1371/journal.pcbi.1002661 (PMC3426562; doi:10.1371/journal.pcbi.1002661)
Supplement: Table S1 — LFA seed residues and associated residue clusters representing independent dynamic segments identified in KIT cytoplasmic region. For each region of the protein, the seed residues identified by the LFA formalism are separated by commas and the associated independent dynamic segments are indicated in parentheses. The analysis was performed on the wild type (WT), mutant D816V (MU) and double mutant D816V/D792E (dbMU). (PDF) [file pcbi.1002661.s003.pdf]

IDS	structural regions	WT	MU	dbMU
S1	JM-P (547-552)	549, 551<br>(547-554)	550<br>(547-553)	549<br>(547-552)
S2	JM-S (560-570)	565<br>(561-569)	564, 566<br>(561-570)	564, 566<br>(561-569)
S3	JM-Z (571-581)	577, 579<br>(574-581)	575<br>(571-577)	574, 579<br>(572-581)
S4	loop I (610-616)	614, 616<br>(588, 609-618)	614<br>(588,609-618)	613, 615<br>(588,608-618)
S5	C-loop-1 (626-647)	628, 631<br>(626-633)	627,631<br>(598-601; 625-635)	628<br>(626-631)
S6	loop II (661-666)	664<br>(585-587; 661-666)	663, 665<br>(586-587; 661-666)	663<br>(586-587; 661-665)
S7	KID (685-694/753-761)	691, 693, 753, 759<br>(688-694; 753-762)	693, 753, 758<br>(684; 687; 690-694; 753-763)	692, 694, 756<br>(687-694; 753-762)
S8	A-loop (810-835)	826, 828<br>(824-831)	819, 828<br>(816-832)	817, 819, 827, 829<br>(814-821) / (824-831)
S9	G-helix (865-885)	872, 877<br>(870-882)	872, 874, 877<br>(870-883)	877, 884<br>(874-887)
S10	C-tail (930-935)	931<br>(926-935)	930<br>(926-935)	929, 931<br>(925-935)

Table S1.
